# A Simplified Analytical Model for Strip Buckling in the Pressure-Assisted Milling Process

**DOI:** 10.3390/ma17153739

**Published:** 2024-07-28

**Authors:** Xuezhi Wang, Kelin Chen, Yanli Lin, Zhubin He

**Affiliations:** State Key Laboratory of High-Performance Precision Manufacturing, School of Mechanical Engineering, Dalian University of Technology, Dalian 116024, China; astart@mail.dlut.edu.cn (X.W.); kchen@dlut.edu.cn (K.C.); linyanli0616@163.com (Y.L.)

**Keywords:** milling, column buckling, post-buckling, Maxwell force, energy barrier, characteristic imperfection

## Abstract

A simplified column-buckling model is developed to understand the buckling mechanism of thin-walled strips restrained by uniform lateral pressure in the milling process. The strip is simplified as two rigid columns connected by a rotation spring, resting on a smooth surface, restrained by a uniform pressure and loaded by an axial force. Two loading cases are considered, i.e., the dead load and the follower load. Analytical solutions for the post-buckling responses of the two cases are derived based on the energy method. The minimum buckling force, Maxwell force and stability conditions for the two cases are established. It is demonstrated that the application of higher uniform pressure increases the minimum buckling force for the column and thus makes the column less likely to buckle. For the same pressure level, the dead load is found to be more effective than the follower load in suppressing the buckling of the system. The effect of initial geometric imperfection is also investigated, and the imperfection amplitude and critical restraining pressure that prevent buckling are found to be linearly related. The analytical results are validated by finite element simulations. This analytical model reveals the buckling mechanism of strips under lateral pressure restraint, which cannot be explained by the conventional bifurcation buckling theory, and provides a theoretical foundation for buckling-prevention strategies during the milling process of thin-walled strips, plates and shells commonly encountered in aerospace or automotive industries.

## 1. Introduction

Thin-walled structures, such as thin-walled metallic strips, plates and shells, are key structural components that are widely used in aerospace, automotive and other industries. To meet the requirements of structural safety and advanced performance of the systems, the thin-walled structures are often required to be manufactured to a specific thickness. Due to the limitations of current manufacturing technologies and/or the challenges of the manufacturing process, the strips, plates or shells of a specific thickness may not be formed successfully, e.g., very thin shells may easily tear during the hydroforming process. To overcome this challenge, thicker strips, plates or shells are adopted to form the part first, and then the thickness of the part is reduced to the specific thickness through the second manufacturing process, i.e., milling [1]. 

**Figure 1:**
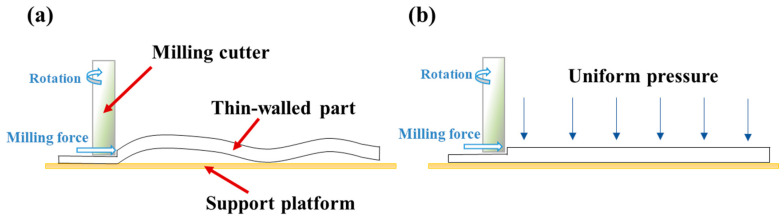
Milling deformation of thin-walled components under (**a**) no pressure-assist; (**b**) pressure-assisted.

It has been observed during the milling process that the thin-walled region in front of the cutter easily shows buckling due to insufficient constraint (see the schematic in Figure 1). As the cutter keeps moving forward, the buckled section would experience a larger amount of thickness reduction or be cut through by the cutter. This leads to failure of the milling process in most cases. 

In order to enhance the stiffness of the thin-walled parts and facilitate the milling process, Lu et al. [2] developed a new technology of vacuum preloading and static pressure support to assist in milling complex surfaces. Tian et al. [3] proposed a new method to suppress vibration and deformation during the mirror milling of thin-walled workpieces by using a magnetic follow-up support fixture. Zheng et al. [4] proposed a multi-point flexible adaptive clamping technology to enhance the local stiffness of large-scale aero-engine casings during milling and drilling processes. This type of strategies usually requires a complex supporting fixture specifically designed for a specific milling task.

It has also been observed that the buckling behavior of the thin-walled part in front of the cutter can be suppressed occasionally by applying lateral pressure to the front region of the cutter. If the buckling is successfully suppressed, the milling process can be successfully performed. However, this buckling-suppression technique may not work if the applied pressure is too small, and thus the milling process cannot be successfully performed due to the buckling of the front section. This phenomenon raises the question of the minimum pressure required to suppress the buckling of a thin-walled section during the milling process, which cannot be answered unless the buckling mechanism of thin-walled structures restrained by a lateral pressure is understood. This study is intended to provide a guideline for the minimum required pressure to suppress the buckling of a strip and ensure the milling process can be successfully performed.

The conventional buckling phenomena do not involve lateral restraining loads such as the lateral concentrated force or the lateral pressure encountered here. Taking the classic Euler buckling problem as an example, when the axial compressive force on a slender column exceeds a critical value, the column buckles. For this class of buckling problem that involves no lateral force restraint, the buckling force can be evaluated using the conventional small-strain bifurcation buckling theory. For another broad class of buckling problems that involve lateral restraint, the lateral restraint can often be simplified as deformable media such as springs that resist tension/compression, bending or torsion. These kinds of bifurcation buckling questions can be handled well by conventional buckling analysis [5].

The buckling response becomes more complicated and the buckling mechanism is unclear when the structure is subjected to lateral load constraints, such as self-weight [6,7], friction force [8], and other external loads [9]. For example, Sadiku and co-authors [10] discussed the problem of optimizing the buckling load of a column considering self-weight and discussed the weight distribution of the column along its height. Virgin and Plaut [11] discussed the critical height of buckling due to self-weight and the bifurcation point when the system is defective. Huang [12] transformed the buckling problem into a nonlinear system of equations by considering the axial force and torque transfer under the coupled action of friction and buckling. Jaculli and Mendes [8] developed a dynamic model to study the effect of the presence or absence of friction on buckling and demonstrated that the difference in friction is a result of the dynamic buckling of the system. Liu [13] developed a buckling model under lateral critical loads based on the Ritz method and investigated the relationship between the variation of parameters and the critical loads. Pi and Bradford [14,15,16,17] theoretically investigated the behavior within linear and nonlinear elastic surfaces as well as the buckling and post-buckling of uniform radial load and established nonlinear equilibrium equations between external load and axial force. The effects of temperature and restraint on the buckling behavior of the structure were also investigated, and the analytical solutions for nonlinear equilibrium paths and ultimate-point buckling load were derived. La Poutré [18] and Zhang [19] studied the elasto-plastic buckling response by concentrating the force and uniform load, respectively. Liu et al. [20] explored the force-induced deformation mechanism for cylindrical shell milling with an ice support, and the ice was modeled as a Winkler support. Bakhach et al. [21] studied the interaction between buckling and lateral buckling of thin-walled beams with arbitrary open sections under axial and lateral loads. Wang [22] investigated the buckling and post-buckling under distributed force on two lateral surfaces and discussed the post-buckling behavior of three different transverse loading approaches based on a multiscale approach.

However, the bifurcation points for such problems cannot be predicted based on traditional bifurcation analysis [23]. In order to address this challenge, various approaches were suggested, e.g., by adding initial geometric defects, introducing dissipation mechanisms, and considering dynamic effects or load eccentricity. Tang et al. [24] studied the buckling strength under uniform lateral load. They proposed an improved analysis-based eigenvalue algorithm considering geometric defects and could predict the vertical load values efficiently and accurately. In the paper by Dou and Pi [25], the influence of geometric imperfections in axially loaded columns with lateral constraints on the inelastic buckling resistance was investigated, and a simplified method for forming critical geometric imperfections in finite element analysis was proposed to obtain the accurate buckling resistance. Gao and Miska [26] applied the principle of virtual work to derive an integrated buckling model. The critical load for the buckling of sinusoidal curves with different friction coefficients was investigated, and it was demonstrated that the system subjected to transverse frictional constraints buckles at a greater compressive force than the frictionless case. Sinira [27] used the Euler–Bernoulli theory to establish the vibration response equations for the buckling and post-buckling configurations of beams with non-classical boundary conditions, and they analyzed the effect of axial loads on the fundamental intrinsic vibration frequency and the dynamic stability of the buckling structure by introducing a small perturbation.

Motivated by the flange wrinkling under a constant blank holding force in the deep-drawing process, Chen and Korkolis [28,29] investigated the buckling of a column constrained by a concentrated force acting at mid-span. It was shown that the buckling behavior of the column differs from that of conventional cases, and the post-buckling response must be established first to identify the critical buckling force for the lateral load-restraint buckling case. Following the spirit of the work of Chen and Korkolis [28,29], here, we investigate the buckling problem of strips constrained by lateral uniform pressure during the milling process. 

It is noted that milling is a very complicated process that involves the complex dynamic interaction of the workpiece and tooling, as well as the damage and fracture of the material. The focus of this manuscript is the establishment of an analytical model that reveals the buckling mechanism of the strip during the pressure-restrained milling process. The complex interaction between the workpiece and tooling is simplified as a force that drives the buckling of the workpiece. As a first step towards understanding this buckling phenomenon, we propose a simplified model of column buckling to unravel the buckling mechanism of strips under lateral uniform pressure constraints. This simplified model greatly facilitates the derivations without the loss of generality of the buckling mechanism. Analytical solutions for the post-buckling responses are derived, and the stability conditions of the structure are analyzed. This study lays a foundation for understanding the buckling behavior of thin-walled strips, plates and shells restrained by lateral loads and provides a guideline for possible buckling-suppression strategies during the milling process, which is expected to improve the quality of thin-walled structures manufactured via milling.

## 2. A Simplified Model of Column Buckling

**Figure 2:**
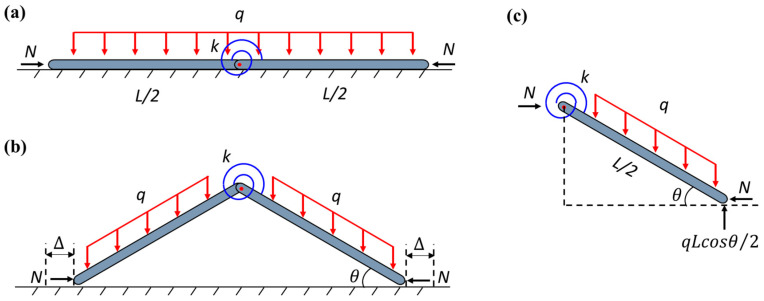
Schematics of Case-1 (dead pressure): (**a**) unbuckled state; (**b**) buckled state; (**c**) buckled free body diagram.

**Figure 3:**
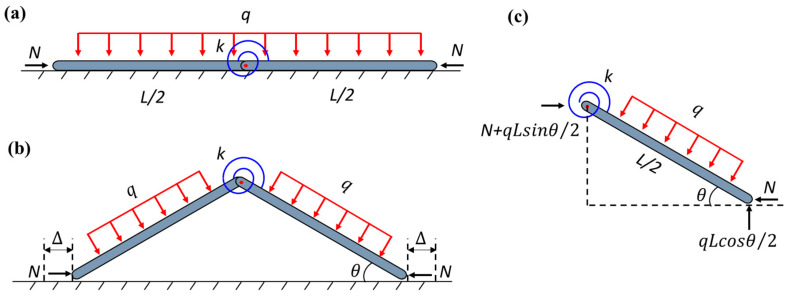
Schematics of Case-2 (follower pressure): (**a**) unbuckled state; (**b**) buckled state; (**c**) buckled free body diagram.

In the conventional milling processes, the thin-walled strips often exhibit weak rigidity and a high removal rate. The buckling of the regions in front of the cutter during the milling process results in uneven wall thickness and an over-tolerance of size and scrap. Therefore, an assisting uniform pressure is applied to the surface of the thin-walled strips to prevent buckling, as shown in Figure 1. To unravel the buckling mechanism of the strip, here, we develop a simplified column-buckling model, which consists of two rigid columns connected by a rotation spring, resting on a smooth surface and initially restrained by uniform lateral pressure. The milling force is denoted as *N*. During the milling processing, two types of pressure can be applied: in Case-1, the load is applied by constantly blowing pressured air downwards from a group of spray nozzles, which is essentially applying a downward dead-load pressure to the column (see Figure 2); in Case-2, the pressure is applied by a gas chamber that ensures the direction of pressure is always perpendicular to the strip, which is essentially a follower load (see Figure 3). Both loading cases are considered in our model. Here, *L/*2 denotes the length of each column, ∆ is the horizontal displacement of the column’s right end, *k* is the stiffness of the rotation spring, and θ is the column’s angle to the surface after buckling. Strips with different widths were used in the actual milling process. The physical pressure applied to the specimens has the unit of Pa. However, to be consistent with the ideal one-dimensional beam model here, the “pressure” q used in our model is a line load (with the unit of N/m), which is essentially the force per unit length along the axial direction of the strip.

To summarize, we propose a simplified column-buckling model to mimic the pressure-assisted strip-buckling behavior during the milling process, and two pressure-loading scenarios are considered. For each loading case, the governing equation and buckling responses will be established below.

### 2.1. Governing Equations

1. Case-1.

Due to the symmetry of the structure, only the right column is considered. According to the force analysis in Figure 2c, the potential energy equation of Case-1 can be expressed as

(1) $$V=\frac{k\theta^{2}}{2}+\frac{qL^{2}}{8}\sin\theta -\frac{NL}{2}\left(1-\cos\theta \right)$$

The normalized potential energy is defined as $\bar{V}=V/k$, the normalized axial force as $\bar{N}=NL/2k\;$, and the normalized uniform pressure as $\bar{q}=qL^{2}/8k$. Equation (1) can be given as

(2) $$\bar{V}=\frac{\theta^{2}}{2}+\bar{q}\sin\theta -\bar{N}\left(1-\cos\theta \right)$$

Using $d\bar{V}/d\theta =0$, the equation for the normalized axial force is

(3) $$\bar{N}=\frac{\theta +\bar{q}\cos\theta }{\sin\theta }$$

2. Case-2.

Different from Case-1, the potential energy equation of Case-2 is expressed as follows:

(4) $$V=\frac{k\theta^{2}}{2}+\frac{qL^{2}}{8}\sin\theta \cos\theta -\frac{NL}{2}\left(1-\cos\theta \right)$$

Following the same normalization manipulations as in Case-1, Equation (4) can be given as

(5) $$\bar{V}=\frac{\theta^{2}}{2}+\frac{\bar{q}\sin2\theta }{2}-\bar{N}\left(1-\cos\theta \right)$$

Using $d\bar{V}/d\theta =0$, the equation for the normalized axial force is

(6) $$\bar{N}=\frac{\theta +\bar{q}\cos2\theta }{\sin\theta }$$

The relationships between the normalized axial force and bucking angle for a given restraining force are established for the two loading cases. It can be seen from Equations (3) and (6) that the axial force is different unless the buckling angle is sufficiently small.

### 2.2. Small-Deflection Analysis

The conventional small-deflection analysis was first conducted in an attempt to identify the buckling force. When the buckling angle θ is very small, Equations (3) and (6) can be linearized as

(7) $$\bar{N}=1+\frac{\bar{q}}{\theta }$$

It can be seen from Equation (7) that for conventional buckling problem without lateral pressure restraint ($\bar{q}=0$), the normalized buckling force is unity. Here, in our problem, the normalized pressure has a finite magnitude, and thus when the buckling angle θ is infinitesimal, the normalized bifurcation axial force approaches infinity. This indicates that the conventional small-deflection analysis is not able to provide a realistic buckling force for this problem, and large-deflection post-buckling analysis must be adopted.

### 2.3. Post-Buckling Response

**Figure 4:**
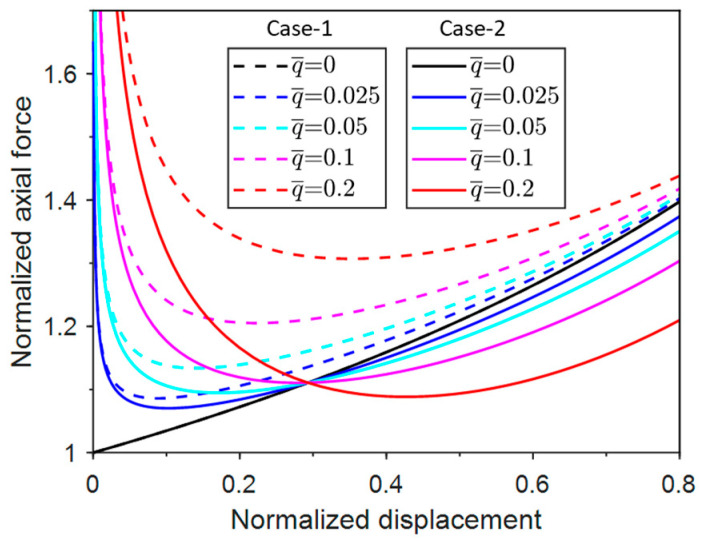
Normalized post-buckling axial force vs. displacement responses for Case-1 and Case-2.

Obtaining the critical buckling force and corresponding buckle configuration of unconventional buckling requires a systematic post-buckling analysis. Therefore, the normalized axial displacement $\bar{\Delta }=2\Delta /L=1-\cos\theta$ is substituted into Equations (3) and (6) to derive the normalized axial force vs. displacement curves of Case-1 and Case-2, as shown in Figure 4. 

It can be seen from Figure 4 that with a certain uniform pressure ($\bar{q}$), the normalized axial force ($\bar{N}$) in Case-1 and Case-2 first decreases to the minimum axial forces (${\bar{N}}_{min}$) and then increases as the displacement increases. The difference between the responses of the two cases becomes more prominent as the pressure increases. It should be noted that when $\bar{q}\;$= 0, the responses of Case-1 and Case-2 overlap, which applies to all related figures in this paper.

### 2.4. Minimum Possible Buckling Force

The minimum possible buckling force for a uniform pressure is the minimum axial force in the post-buckling regime. When the normalized axial force is less than the minimum axial force, the non-trivial buckled equilibrium condition cannot be satisfied, and thus buckling is impossible.

The equation of ${\bar{N}}_{min}$ for Case-1 can be rewritten as

(8) $${\bar{N}}_{min}=\frac{\theta_{min}+\bar{q}\cos\theta_{min}}{\sin\theta_{min}}$$

Using $d\bar{N}/d\bar{\Delta }=0$ and Equation (3), we have

(9) $$\frac{d\left(\frac{\theta +\bar{q}\cos\theta }{\sin\theta }\right)}{d\left(1-\cos\theta \right)}=0$$

The simplified equation is

(10) $$\sin\;\theta -\theta \cos\theta -\bar{q}=0$$

The approximate solution of θ in Equation (10) is derived from the Taylor series expansion θ=3q¯13, and ${\bar{N}}_{min}$ can be expressed as

(11) N¯min≈1+3q¯232−113q¯43120−33q¯2560

Only taking the first term of $\bar{q}$, the equation is

(12) N¯min≈1+3q¯232

Similarly, the$\;{\bar{N}}_{min}$ of Case-2 can be rewritten as

(13) $${\bar{N}}_{min}=\frac{\theta_{min}+\bar{q}\cos2\theta_{min}}{\sin\theta_{min}}$$

The approximate solution is also θ=3q¯13, and Equation (13) becomes

(14) N¯min≈1+3q¯232−713q¯43120+673q¯2560

Only taking the first term of $\bar{q}$, the minimum force is approximated as

(15) N¯min≈1+3q¯232

**Figure 5:**
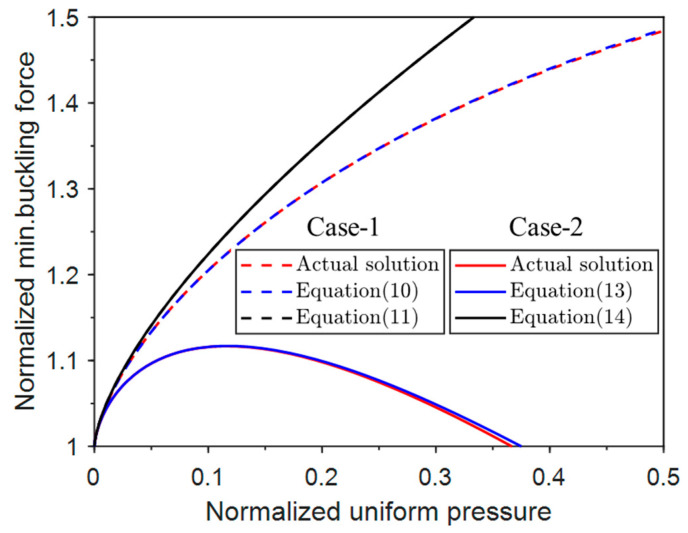
Normalized minimum possible buckling forces for Case-1 and Case-2.

Figure 5 shows the variation of the minimum axial force with the normalized uniform pressure for the two cases. The minimum axial force of Case-1 increases with the increase in the normalized uniform pressure. In contrast, the minimum axial force of Case-2 increases and then decreases with the increase in the normalized uniform pressure. There is a maximum ${\bar{N}}_{min}$ when $\bar{q}\;$= 0.1 (for Case-2 only). By comparing the approximate values with the actual values, it can be seen that the approximation values of the four terms for both cases are in better agreement with the actual values. The approximation of two terms is a reasonably good approximation for Case-1 but a poor approximation for Case-2. Another important observation is that the value of the minimum axial force of Case-1 is larger than that of Case-2, indicating that the system of Case-2 is more prone to buckle than that of Case-1. This can be attributed to the horizontal component of the follower load that facilitates post-buckling.

### 2.5. Maxwell Force

The Maxwell Force (${\bar{N}}_{mx}$) is often used for the determination of critical load when several different configurations with the same potential energy coexist.

**Figure 6:**
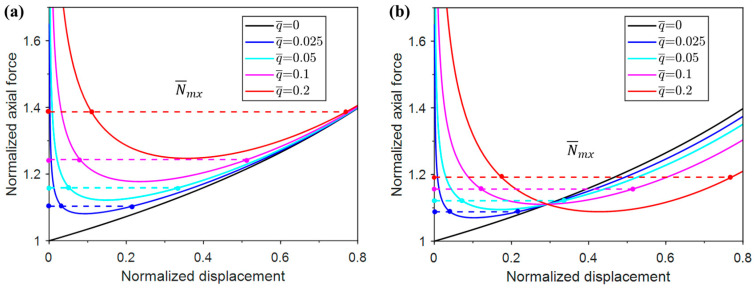
The constructed Maxwell forces according to the post-buckling axial force vs. displacement responses under different normalized pressure levels: (**a**) Case-1; (**b**) Case-2.

For the present buckling problem, the Maxwell force indicates that for a certain axial force, the unbuckled and buckled structures exhibit the same potential energy, i.e., the area below the force vs. displacement curve is equal to that below the horizontal dashed line (see Figure 6). Thus, the Maxwell force equation of Case-1 is

(16) $$\overset{{\bar{\Delta }}_{mx}}{\underset{0}{\int }}\bar{N}d\bar{\Delta }={\bar{N}}_{mx}{\bar{\Delta }}_{mx}$$

The simplification leads to

(17) $$\frac{\theta_{mx}^{2}}{2}+\bar{q}\sin\theta_{mx}=\frac{\theta_{mx}+\bar{q}\cos\theta_{mx}}{\sin\theta_{mx}}\left(1-\cos\theta_{mx}\right)$$

The approximate solution is θ=12q¯13, and ${\bar{N}}_{mx}$ can be expressed as

(18) N¯mx≈1+12q¯234−12q¯43120+12q¯25040

Only taking the first term of $\bar{q}$, the ${\bar{N}}_{mx}$ equation can be rewritten as

(19) N¯mx≈1+12q¯234

Similarly, the $\;{\bar{N}}_{mx}$ equation of Case-2 is

(20) $$\frac{\theta_{mx}^{2}}{2}+\frac{\bar{q}\sin2\theta_{mx}}{2}=\frac{\theta_{mx}+\bar{q}\cos2\theta_{mx}}{\sin\theta_{mx}}\left(1-\cos\theta_{mx}\right)$$

The approximate solution is θ=12q¯13, and the equation is

(21) N¯mx≈1+12q¯234−212q¯4315+31712q¯210080

Only taking the first term of $\bar{q}$, the equation is

(22) N¯mx≈1+12q¯234

**Figure 7:**
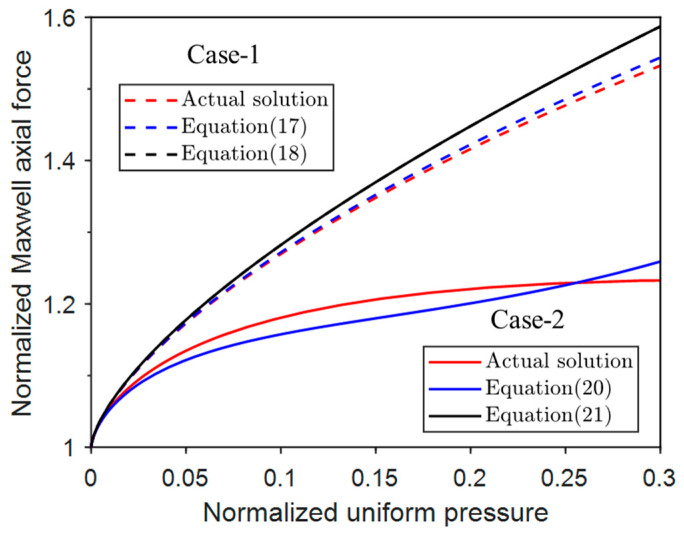
Normalized Maxwell forces for different restraining pressures.

Figure 6 shows that the Maxwell force, which is denoted by dashed line with the same color as the corresponding solid line, increases with the given normalized uniform pressure levels for both Case-1 and Case-2. The evolution of $\;{\bar{N}}_{mx\;}$ with the normalized pressure $\bar{q}$ is plotted in Figure 7. It can be seen that $\;{\bar{N}}_{mx\;}$ of Case-1 is always larger than that of Case-2 for all the pressure levels, and the difference increases with the increase in the pressure.

Figure 7 also compares the approximations of $\;{\bar{N}}_{mx\;}$ for Case-1 and Case-2. The four-term approximation of the equation is closer to the actual value. The approximation of $\;{\bar{N}}_{mx\;}$ with two-term approximation is acceptable for Case-1 but poor for Case-2.

### 2.6. Potential Energy

In practice, the axial loading systems can be controlled either by displacement or by force. Therefore, it is worth investigating the potential energy of both cases.

1. Displacement-control case.

The potential energy of the buckled configuration with the displacement control of Case-1 from Equations (2) and (3) is

(23) $$\bar{V}=\frac{\theta^{2}}{2}-\frac{\left(\theta -\bar{q}\right)\left(1-\cos\theta \right)}{\sin\theta }$$

Similarly, the potential energy of Case-2 from Equations (5) and (6) is

(24) $$\bar{V}=\frac{\theta^{2}}{2}-\frac{2\left(\theta -\cos\theta \right)+\bar{q}\left(2\cos\theta -2\cos2\theta \cos\theta -\sin2\theta \sin\theta \right)}{\sin\theta }$$

**Figure 8:**
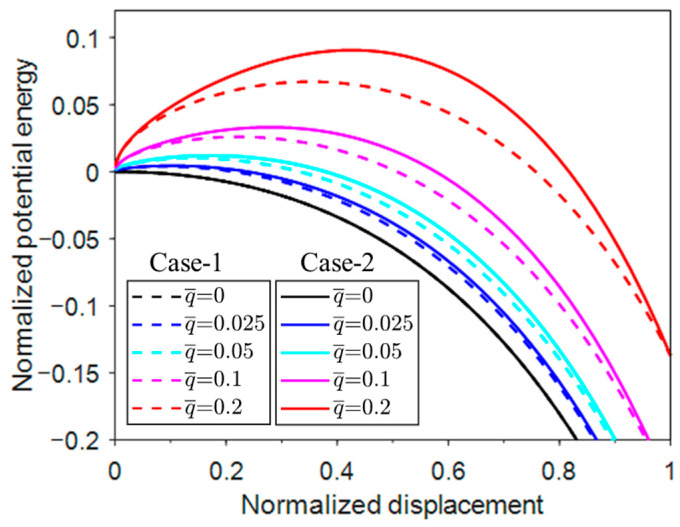
Normalized potential energy vs. displacement under different normalized uniform pressures.

The potential energy-variation curves for normalized axial displacement at different pressure levels are shown in Figure 8.

It can be seen that when $\bar{q}$ = 0, i.e., the conventional buckling case, the potential energy is maximum at $\bar{\Delta }$ = 0 and then decreases, and for $\bar{q}\neq \;$0, the potential energy of the system is generally higher than that of conventional buckling and has a maximum value at a certain $\bar{\Delta }\;\neq \;$0. For a given displacement, the potential energy of Case-1 is smaller than that of Case-2, and the potential energy evolves with a similar trend for Case-1 and Case-2.

2. Force-control case.

The equilibrium condition corresponds to a given axial force for which multiple equilibrium configurations are possible in the case of force-controlled buckling. 

**Figure 9:**
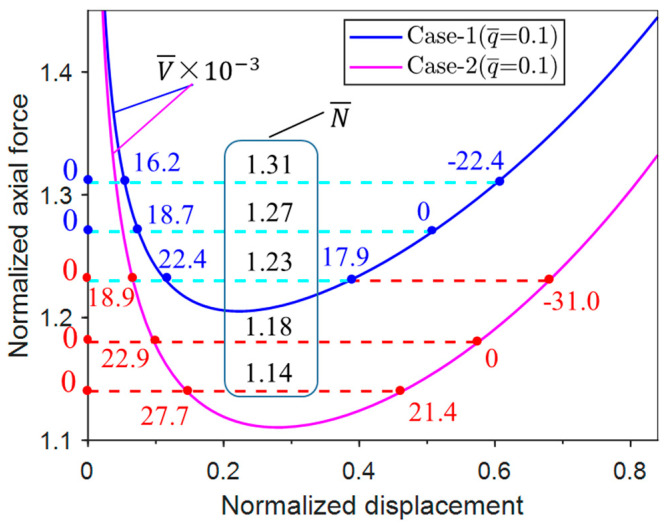
Normalized potential energies of Case-1 and Case-2 in different equilibrium configurations under a group of axial-force control cases with given normalized pressure of 0.1.

As shown in Figure 9, for a given value of both the normalized axial force and the normalized uniform pressure, axial forces smaller than $\;{\bar{N}}_{min}$(=1.205) exist only for the trivial equilibrium case, and thus buckling does not happen. When axial forces are larger than ${\bar{N}}_{min}$, there are two non-trivial configurations: compared to the trivial case, the first non-trivial configuration that corresponds to a smaller axial displacement possesses a higher potential energy, and the second non-trivial configuration that corresponds to a larger axial displacement has less energy compared to the first non-trivial configuration. From the energy viewpoint, the energy of first non-trivial configuration is always greater than the trivial configuration and the second non-trivial configuration, indicating that an energy barrier is always present that prevents buckling from occurring. For the same $\bar{N}$ case, the energy of the first non-trivial configuration of Case-1 is greater than that of Case-2, and thus the energy barrier of Case-1 is greater.

### 2.7. Stability and Energy Barrier

The potential energy can be given as:

(25) $$\bar{V}=\overset{\bar{\Delta }}{\underset{0}{\int }}\bar{N}d\bar{\Delta }-\bar{N}\;\bar{\Delta }$$

As long as the pressure is not zero, the first variation of the potential energy from Equations (2) and (5) for both cases are positive ($\delta \bar{V}=\bar{q}\delta \theta >0$), so the trivial configuration is the one which is stabilized. The two non-trivial cases are analyzed using the second derivative of potential energy from Equation (25):

(26) $$\frac{d^{2}\bar{V}}{d{\bar{\Delta }}^{2}}=\frac{d\bar{N}}{d\bar{\Delta }}$$

The first non-trivial equilibrium configuration (left equilibrium configuration) is an unstable state ($d^{2}\bar{V}/d{\bar{\Delta }}^{2}<0$), while the second non-trivial configuration (right equilibrium configuration) is a stable state ($d^{2}\bar{V}/d{\bar{\Delta }}^{2}>0$), as shown in Figure 9. Therefore, in contrast to the infinitesimal energy perturbation that are necessary for conventional case, a finite energy perturbation is required for buckling of the system, which must be no less than the potential energy of the first non-trivial configuration. Because the energy of the unstable configuration is higher than the stable configuration, when the system switches from unstable to stable case, it will release a certain amount of energy. The Maxwell correspondence is such that the first jump releases the same amount of external perturbation energy as the second one, so the net energy exchange is zero. In contrast, the potential energy of Case-1 is larger than that of Case-2, indicating that Case-1 requires a larger amount of perturbation energy to snap through to the stable state.

**Figure 10:**
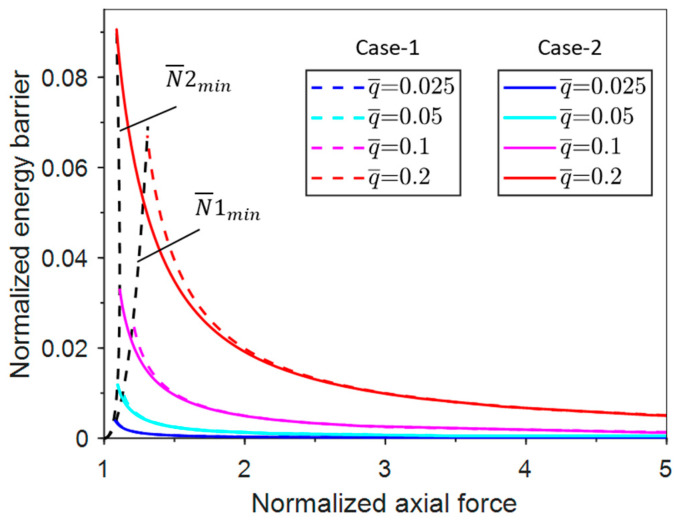
Variation of normalized buckling energy barrier vs. axial force. under different uniform pressures.

The energy analysis above shows that energy barrier exists for this unconventional buckling problem. The variation of the energy barrier with normalized axial force under various pressure levels is illustrated in Figure 10. There is a maximum energy barrier for a specific axial force ($\;{\bar{N}}_{min}$) for each pressure level. The energy barrier decreases to zero with the normalized axial force increasing to infinity. The energy barriers of Case-1 and Case-2 both increase monotonically with the increasing normalized uniform pressure.

**Figure 11:**
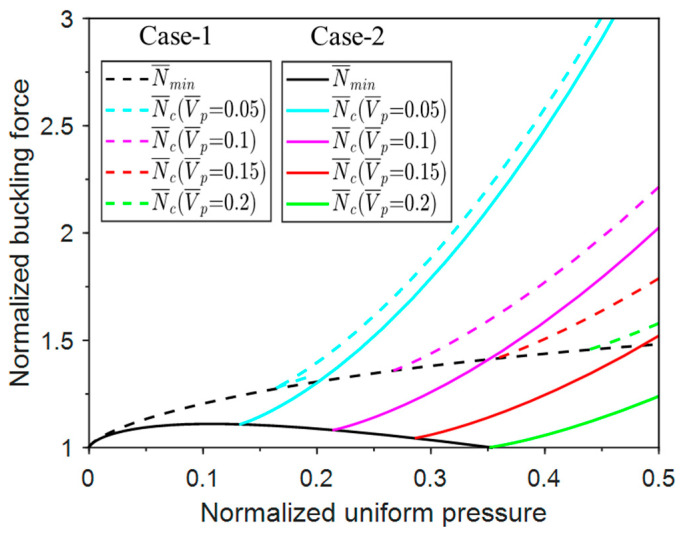
Variation of the normalized buckling force vs. the uniform pressure under a group in perturbation energy levels.

The buckling force is dependent on the energy perturbation of the external agency, and the energy barrier is also dependent on the axial force. For a given axial force, there exists a corresponding energy barrier, and the column buckles when the perturbation energy is greater than this energy barrier. Thus, the buckling force is dependent on when perturbation energy is supplied and the amount of supplied energy. As shown in Figure 11, the buckling force varies with the normalized uniform pressure at various perturbation energy levels. For a certain perturbation energy, the buckling force of Case-1 increases along with pressure. As the perturbation energy increases, the buckling force decreases. Whenever the energy is larger than the maximum energy barrier corresponding to$\;{\bar{N}}_{min}$, the column is prone to buckle at ${\bar{N}}_{min}$.

For example, in the case of a perturbation energy level of 0.05, Case-1 will buckle at ${\bar{N}}_{min}$ for a normalized uniform pressure of less than 0.15. At the same perturbation energy level, Case-2 will buckle at ${\bar{N}}_{min}$ for a normalized uniform pressure less than 0.12. When the perturbation energy level increases to 0.1, Case-1, with a pressure of less than 0.25, can buckle at$\;{\bar{N}}_{min}$, while Case-2, with a uniform pressure less than 0.22, can buckle at$\;{\bar{N}}_{min}$. As the perturbation energy increases to 0.15 and 0.2, the pressure ranges of Case-1 and Case-2 in which the column can buckle at$\;{\bar{N}}_{min}$ expand to approximately 0.35, 0.28 and 0.45, 0.35, respectively. 

## 3. Imperfection Sensitivity

### 3.1. Characteristic Imperfection

The existence of initial geometries or material imperfections affects the buckling force and post-buckling responses. Therefore, studying the imperfection sensitivity of the systems is useful for the design of structures [27].

**Figure 12:**
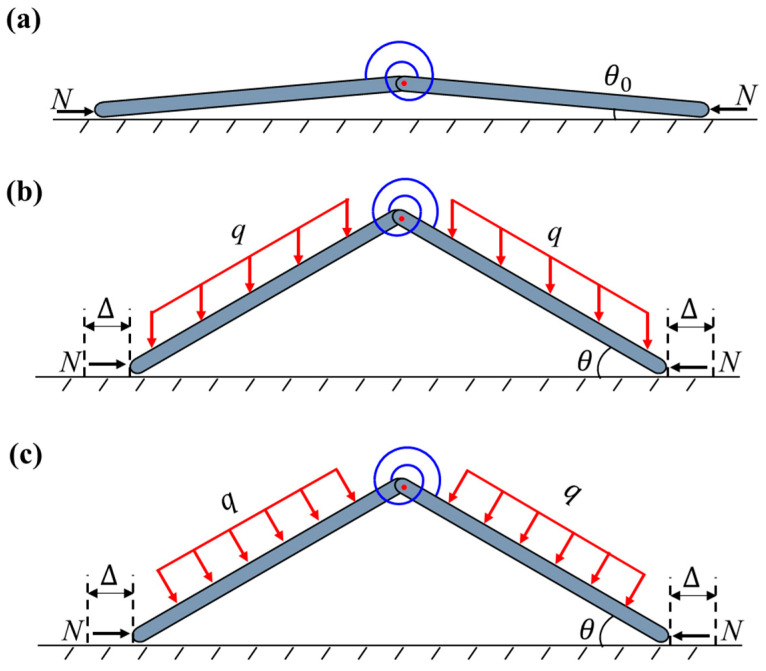
Schematic of the buckling configuration with imperfection. (**a**) initial state; (**b**) buckled state of Case-1; (**c**) buckled state of Case-2.

The initial geometric imperfection in this problem is considered to be a small angle ($\theta_{0}$) initially formed at the end between the rigid column and the horizontal plane, after a uniform pressure (q) is applied laterally and an axial force (*N*) is applied at both ends. After the system buckles, the column and the plane form an angle (θ), as shown in Figure 12.

The post-buckling potential energy of Case-1 is

(27) $$\bar{V}=\frac{{\left(\theta -\theta_{0}\right)}^{2}}{2}+\bar{q}\sin\theta -\bar{N}\left(\cos\theta_{0}-\cos\theta \right)$$

Using $d\bar{V}/d\theta =0$, the equilibrium condition is obtained as

(28) $$\theta -\theta_{0}+\bar{q}\cos\theta -\bar{N}\sin\theta =0$$

In this case of the normalized axial displacement $\bar{\Delta }=\cos\theta_{0}-\cos\theta$, Equation (28) is linearized through the assumption that the deformation angle θ is very small, which leads to

(29) $$(1-\bar{N})\theta =\theta_{0}-\bar{q}$$

Similarly, the potential energy of Case-2 is

(30) $$\bar{V}=\frac{{\left(\theta -\theta_{0}\right)}^{2}}{2}+\frac{\bar{q}\sin2\theta }{2}-\bar{N}\left(\cos\theta_{0}-\cos\theta \right)$$

Using $d\bar{V}/d\theta =0$, the equilibrium condition is

(31) $$\theta -\theta_{0}+\bar{q}\cos2\theta -\bar{N}\sin\theta =0$$

Equation (31) is linearized through the assumption that the deformation angle θ is very small, which leads to

(32) $$(1-\bar{N})\theta =\theta_{0}-\bar{q}$$

When $\theta_{0}-\bar{q}=0$, the equilibrium equation becomes an eigenvalue equation:

(33) $$(1-\bar{N})\theta =0$$

Equation (33) corresponds to the eigenvalue problem of the conventional buckling in the absence of constraint pressure and imperfection. Therefore, $\theta_{0}=\bar{q}$ is considered as the characteristic imperfection amplitude of the system, which balances the effect of the restraining pressure on the buckling force.

The same characteristic imperfection is obtained for Case-1 and Case-2. The normalized characteristic imperfection amplitude can be defined as ${\bar{\theta }}_{0}=\theta_{0}/\;\bar{q}$, after we obtain the characteristic imperfection amplitude $\theta_{0}=\bar{q}$. When ${\bar{\theta }}_{0}=1$, the bifurcation axial force of Case-1 is

(34) $$\bar{N}=\frac{\theta +\bar{q}\left(\cos\theta -1\right)}{2}$$

Using $d\bar{V}/d\bar{\Delta }=0$, the post-buckling equilibrium condition from Equations (26) and (34) can then be established as

(35) $$\sin\;\theta -\theta \;\cos\theta -\bar{q}\left(\cos\theta -1\right)=0$$

Similarly,${\bar{\theta }}_{0}=1$ leads to the bifurcation axial force of Case-2

(36) $$\bar{N}=\frac{\theta +\bar{q}\left(\cos2\theta -1\right)}{2}$$

Using $d\bar{V}/d\bar{\Delta }=0$, the equation from Equations (30) and (36) is

(37) $$\sin\;\theta -\theta \;\cos\theta -\bar{q}\left(\cos2\theta -1\right)=0$$

**Figure 13:**
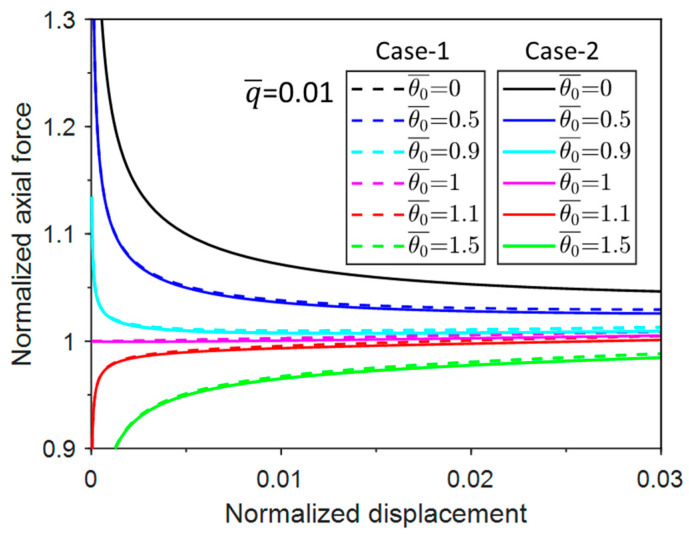
Normalized post-buckling axial force vs. displacement responses with normalized uniform pressure of 0.01 for a group of normalized imperfection amplitudes.

Figure 13 shows the responses of the post-buckling axial force with displacement when $\bar{q}=0.01$. When ${\bar{\theta }}_{0}\;$reaches 1 (i.e., the characteristic imperfection amplitude), this is a bifurcation problem as the normalized buckling axial force increases from 1. The axial force increases from zero to a level lower than the bifurcation response with increasing imperfection amplitude ${\bar{\theta }}_{0}$. The trends of Case-1 and Case-2 are essentially the same. Overall, the responses of all imperfect responses approach the bifurcation response from above (amplitude < 1) or from below (amplitude > 1). Therefore, the bifurcation response is analogous to the traditional buckling response and can be used as a reference for the imperfection responses. 

This essentially establishes the linear relationship between the imperfection amplitude and the critical holding pressure to prevent buckling. In real-world cases where more complex interactions are involved, e.g., when the imperfection magnitude is too large or plastic deformation occurs, the linear relationship may not hold.

**Figure 14:**
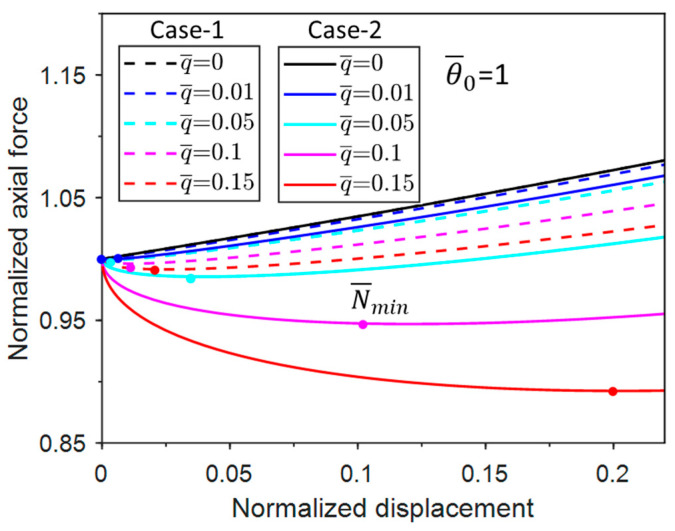
Normalized post-bifurcation axial force vs. displacement responses with the characteristic imperfection amplitude for a group of normalized uniform pressure.

Figure 14 shows the post-bifurcation responses with a range of pressure levels. As the pressure increases, there is a decrease in the normalized axial force and a corresponding decrease in the minimum axial force. The same trend can be observed for Case-1 and Case-2. Nevertheless, the minimum axial force in Case-2 is much smaller than that of Case-1.

### 3.2. Asymmetric Bifurcation

There exists a bifurcation of the structure at the critical buckling force with characteristic imperfection amplitude. Buckling occurs only at the top of the plane because of the constraint of the rigid plane. In fact, the structure could bifurcate either to the up- or downside if there is no rigid plane. As a result, it is important to analyze the buckling and post-bifurcation responses both upward and downward.

**Figure 15:**
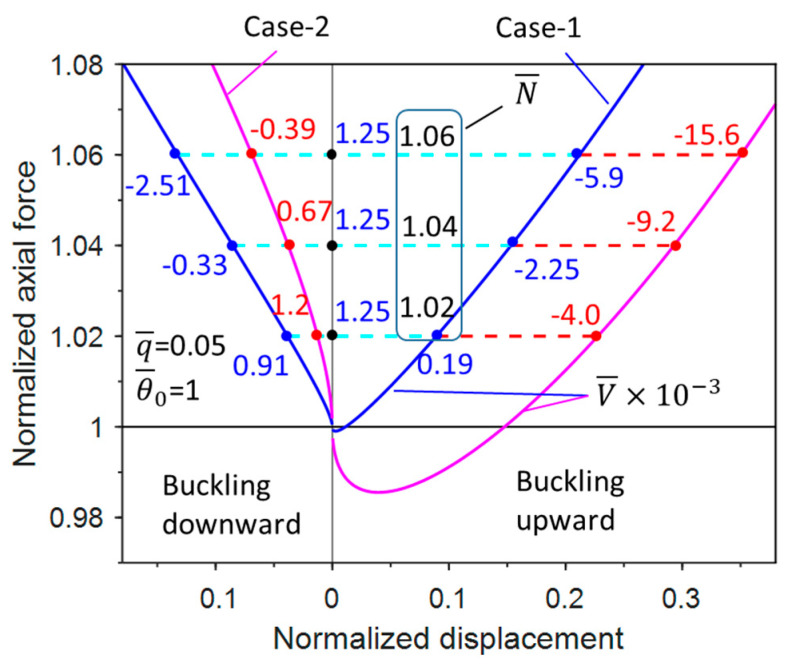
Normalized potential energy under various equilibrium configurations for three axial-force control cases.

Figure 15 shows the bifurcation responses of Case-1 and Case-2 obtained from Equations (34) and (36) for $\bar{q}=0.05$ and ${\bar{\theta }}_{0}=1$, as well as the potential energy for each configuration. The right-hand side indicates upward buckling and the left-hand side indicates downward buckling. Both Case-1 and Case-2 show asymmetric bifurcations. While the potential energy obtained by Equations (27) and (30) differs significantly between the two modes, the normalized potential energy in the trivial case stays with a value equal to 1.25 × 10^−3^, indicating that the energy stored within the spring during the preloading of the column is the global maximum in the trivial case, and the trivial case is thus always unstable. Since upward buckling has global minimum values and downward buckling has local minimum values, upward buckling is globally stable and downward buckling is locally stable. It is interesting to note that for upward buckling, Case-2 is more prone to buckle than Case-1; for downward buckling, however, Case-1 is more likely to buckle than Case-2, because the horizontal component of the follower load in Case-2 tends to prevent the evolution of post-buckling configuration.

## 4. Finite Element Simulations

### 4.1. FE Model

**Figure 16:**
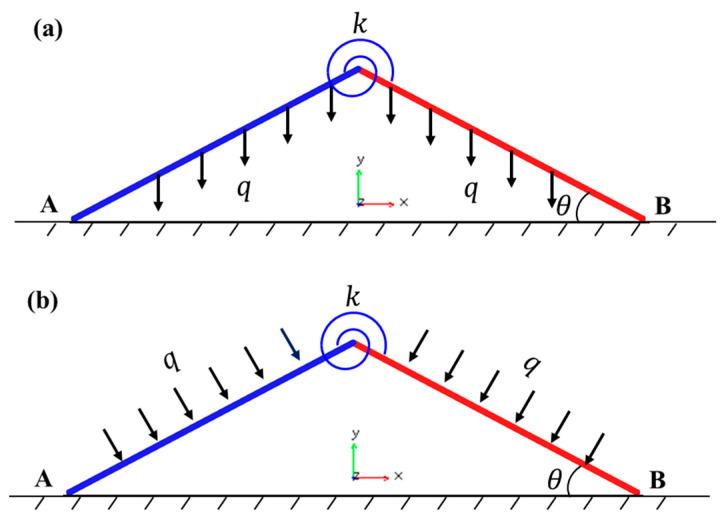
Finite element model of (**a**) Case-1 (dead load); (**b**) Case-2 (follower load).

In order to validate the developed analytical model, a finite element model of column buckling was established using the commercial software Adams 2020. The column selected was a 1 mm × 1 mm× 100 mm rigid column with a density of *ρ* = 7.8 g/cm^3^, and the spring stiffness was defined as *k* = 1 N·mm/°. The upper surface of the column was subjected to normalized uniform pressure (both the dead-load and follower-load cases were simulated), and the column exhibited an initial imperfection at an inclined angle of 1° to the ground. The two ends A and B were allowed to move only in the horizontal direction, as shown in Figure 16.

The structural responses of the two cases were extracted from the FE models and compared with those from the analytical solutions.

### 4.2. Numerical Results 

**Figure 17:**
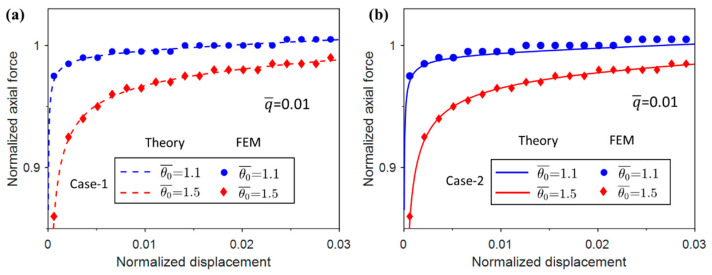
Comparison of the normalized axial force vs. displacement from theory and FEM for different imperfection amplitudes. (**a**) Case-1; (**b**) Case-2.

The normalized axial force vs. displacement at end B was obtained and is plotted together with the analytical force vs. displacement responses of Case-1 and Case-2 in Figure 17.

It can be seen from Figure 17 that the numerical and theoretical solutions are basically the same for different imperfections, which validates the previous analysis. The numerical solution of the normalized axial force for ${\bar{\theta }}_{0}=1.5$ is overall more accurate than ${\bar{\theta }}_{0}=1.1$. This is due to the fact that the initial imperfection in the simulation is too small and there are some uncertainties when processing the numerical results. The uncertainties are smaller as the imperfection amplitude becomes larger.

## 5. Pressure Restraint vs. Force Restraint

**Figure 18:**
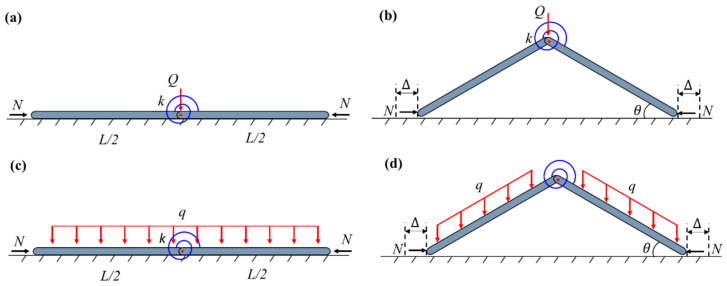
Schematics of concentrated force case: (**a**) unbuckled configuration; (**b**) buckled configuration and constant pressure case; (**c**) unbuckled configuration; (**d**) buckled configuration.

The constant pressure restraint is investigated in this paper because the pressure is adopted to in our milling process to prevent the buckling of the strip. Clearly, pressure is not the only option for buckling suppression. In practice, a constant force can also be used as a load restraint to prevent buckling. For example, the constant blank holding force is often used during the deep-drawing process to prevent wrinkles in the cups [30]. It is interesting to compare the buckling-prevention capabilities of the pressure and concentrated force, under the premise that the total restraining forces of the two loads are the same. To this end, the same structure that is subjected to concentrated force constraint in the middle was analyzed (Case-CF) [28] and compared with the pressure-restrained case, as schematically shown in Figure 18.

Assuming that the same level of total constraining force is applied, i.e., *Q* = *qL*=$\;P_{0}$, the normalized post-buckling axial force vs. displacement responses of the pressure restraint (Case-1 and Case-2) and concentrated force restraint (Case-CF) are established.

**Figure 19:**
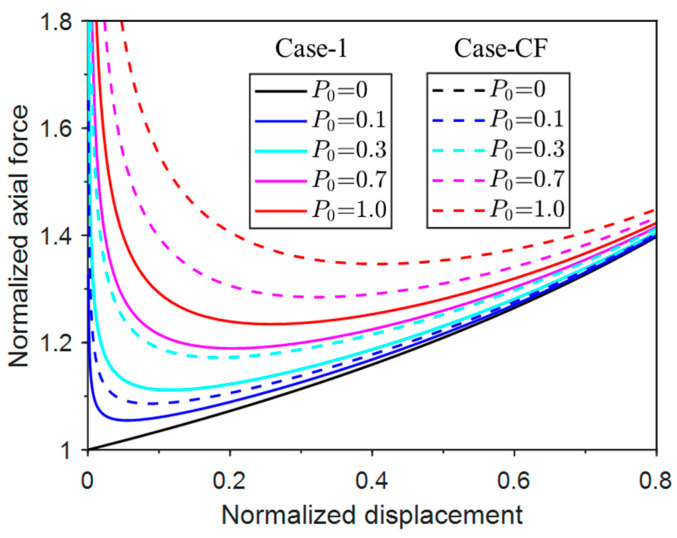
Normalized post-buckling axial force vs. displacement responses of Case-1 and Case-CF.

**Figure 20:**
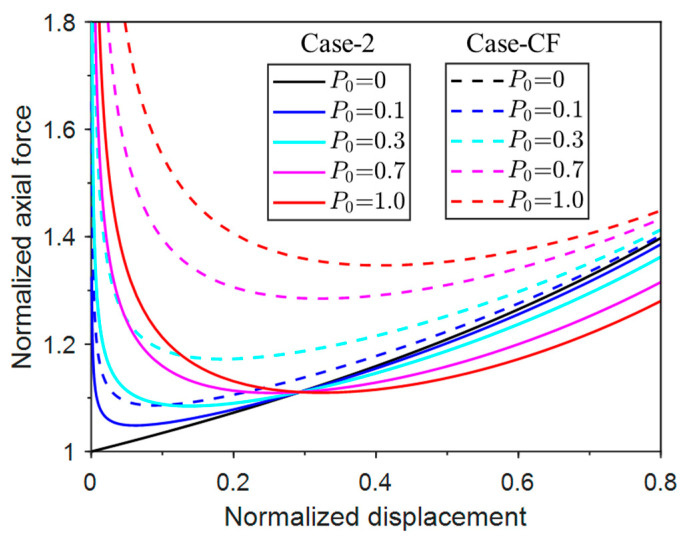
Normalized post-buckling axial force vs. displacement responses of Case-2 and Case-CF.

Figure 19 compares the responses of Case-1 and Case-CF. It can be seen that the axial force of Case-CF is always larger than the values of Case-1 when the net force $P_{0}$ is the same, which indicates that a larger buckling force is generally required to reach the post-buckling equilibrium, and thus buckling is more difficult to trigger for the concentrated-force restraint case. The same conclusion can be reached in Figure 20, where the responses of Case-2 and Case-CF are compared. The axial force differences of the two cases are even larger. The two comparisons indicate that the concentrated force restraint is more effective in buckling prevention than the other two pressure restraint cases for this column-buckling problem at hand. However, due to the uncertainty of the buckling wavelength and wave peak in the milling process, the optimal location of the applied concentrated force cannot be determined a priori. Therefore, the uniform pressure restraint is adopted in our milling process for practical reasons.

## 6. The Critical Pressure to Prevent Buckling

In the actual milling process, the milling force can be measured. It is beneficial to identify the critical pressure level above which the strip will not buckle. This critical pressure is thus a lower bound of the buckling-prevention pressure. For Case-1, two axial loading categories must be considered:

1. When the milling force $\bar{N}\leq 1.57$.

**Figure 21:**
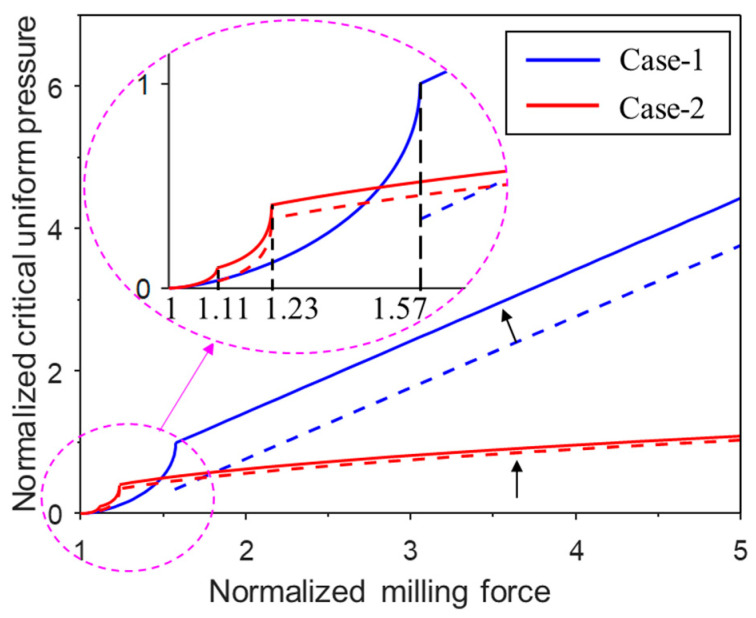
The normalized critical pressure vs. milling force responses for Case-1 and Case-2.

When the milling force is below 1.57, it can be shown that for a given milling force, there is a critical pressure, above which a post-buckling equilibrium cannot be satisfied for any normalized axial displacement between 0 and 1. In such a case, no matter how much perturbation energy is provided, the buckled configuration will resume the straight configuration. The milling force essentially corresponds to the minimum possible buckling force for the given pressure restraint. This way, the critical pressure is identified for any given milling force, as shown in Figure 21 by the solid blue line (for $\bar{N}\leq 1.57$). 

It can be seen from Figure 21 (solid blue line) that the critical pressure increases drastically with the milling force. For Case-1, when the milling force is 1.57, the critical pressure is 1, and the post-buckling equilibrium configuration corresponds to an axial displacement of 1, which means the two ends of the column meet and the two columns are vertical. Assuming there is no penetration of the two ends, this case of $\bar{N}=1.57$ is the limiting case of our minimum possible buckling-force analysis.

2. When the milling force $\bar{N}>1.57$.

When the milling force is above 1.57, it can be shown that for a given milling force, there is always a post-buckling equilibrium at a certain normalized axial displacement between 0 and 1, no matter how much pressure is applied. Therefore, there is no solution for the buckling-prevention pressure. If a sufficient amount of perturbation energy is provided, the structure may snap through to the collapsed case, i.e., $\bar{\Delta }$ = 1. The straight case has a lower potential energy compared to the collapsed case from the energy viewpoint, as long as the milling force does not exceed the Maxwell force. In other words, when

(38) $$\bar{q}\geq \bar{N}-\frac{\pi^{2}}{8}$$

which is derived from Equation (2) by setting θ=π/2 and $\bar{V}=0$, and the straight case will be globally stable. This provides a preliminary guidance on how the pressure should be increased with the increase in milling force, given that a rigorous lower bound for the buckling-prevention pressure does not exist. Equation (38) is plotted in Figure 21 as the blue dashed line. However, it can be noted that there is a discontinuity at $\bar{N}=1.57$, which is due to the different mechanisms used to identify the critical pressure. Considering that the applied pressure should be a continuous function of the milling force, the blue dashed line for Equation (38) is shifted up by 0.66 ($\;\bar{q}=1.0$) to satisfy the pressure continuity condition at $\bar{N}=1.57$, as plotted in Figure 21 as the blue solid line. Since the pressure is increased, the energy barrier increases, and the structure becomes less likely to buckle. Therefore, the identified critical pressure is more conservative, which serves as physically based empirical guidance for buckling prevention, given that a rigorous solution for buckling-prevention pressure does not exist.

Following a similar analysis as in Case-1, the critical pressure for Case-2 can be explored. However, three axial loading regimes must be considered for Case-2:

1. When the milling force $\bar{N}\leq 1.11$

When the milling force is below 1.1, there is a critical pressure, above which a post-buckling equilibrium is not possible for any normalized axial displacement between 0 and 1. The milling force is just the minimum possible post-buckling force for a given pressure restraint. The critical pressure that prevents buckling is identified for any given milling force, as shown in Figure 21 by the solid red line.

2. When the milling force $1.11\leq \bar{N}\leq 1.23$.

When the milling force is between 1.11 and 1.23, the post-buckling equilibrium always exists at a certain normalized axial displacement between 0 and 1 for any pressure level. Therefore, the buckling-prevention pressure does not exist. However, the straight case will be globally stable from the energy viewpoint as long as the milling force is less than the Maxwell force. The relationship between the Maxwell force and the critical pressure can be obtained from Equation (20), which is plotted in Figure 21 as the red dashed line.

3. When the milling force $\bar{N}\geq 1.23$.

Due to the follower loading in Case-2, the potential energy contribution from the pressure is zero when the structure collapses (θ=π/2 in Equation (5)). Therefore, when the milling force is greater than 1.23, the potential energy of the collapsed case is always negative, i.e., the straight case is locally stable, and Maxwell force does not exist for $\bar{N}\geq 1.23$. The idea of adopting Maxwell force for buckling prevention no longer works. To provide a guideline for buckling prevention, we note that at the critical state of $\bar{N}=1.23$, the normalized energy barrier for the snap through from the straight case to the collapsed case is 122 × 10^−3^. When the milling force exceeds this value, we assume the pressure should increase accordingly to at least maintain this level of energy barrier, so that the snap through of the locally stable straight case to the collapsed case is conditionally prevented. Using Equations (5) and (6), the critical pressure corresponding to the energy barrier of 122 × 10^−3^ for a given milling force above 1.23 can be obtained. Following a similar analysis as in Case-1, to avoid the discontinuity at $\bar{N}=1.23$, the dashed red line for $\bar{N}\geq 1.11$ is shifted up by 0.066 ($\bar{q}=0.403$) to satisfy the pressure continuity condition at $\bar{N}=1.23$, as plotted in Figure 21 as the red solid line. The new line provides more conservative guidance for buckling prevention based on Maxwell force (for $1.11\leq \bar{N}\leq 1.23$) and energy barriers (for $\bar{N}\geq 1.23$).

## 7. Conclusions

Motivated by the buckling behavior of thin-walled strips during the pressure-assisted milling process, this paper investigates the buckling mechanism of the pressure-restrained buckling behavior of strips. A simplified buckling model is established to reveal the buckling mechanism of such an unconventional buckling phenomenon, which comprises of two rigid columns connected by a spring and restrained by uniform dead or follower pressure on a smooth surface. Using this model, the post-buckling responses and stabilization conditions of the systems under dead- and follower-pressure loadings are established and compared. The influence of initial geometric imperfections on the buckling behavior of the system is also investigated and validated by numerical simulations. Below are the main conclusions from this work:

(1) The application of pressure to the system provides an energy barrier that prevents the bifurcation buckling of the system.
(2) For a given pressure level, the dead pressure is more effective than the follower pressure in preventing the buckling of the system.
(3) Establishing the post-buckling response of the system is crucial for the estimation of the buckling force. The buckling force is dependent on the applied pressure and external perturbation energy.
(4) A critical imperfection amplitude can be identified for this system, which can be utilized to convert this pressure-restrained unconventional buckling problem into a conventional bifurcation buckling problem without pressure restraint.
(5) Depending on the magnitude of the milling force, the critical pressure to prevent buckling can be provided based on one of the three different mechanisms: the minimum possible post-buckling force, the Maxwell force, and the energy barrier.

This work is a first step towards understanding the buckling mechanism of thin strips in the pressure-assisted milling process, which lays a theoretical foundation for the future work of establishing the post-buckling responses of the actual metal strips under pressure restraint and efficient buckling-suppression strategies in the milling process.

## Data Availability

The original contributions presented in the study are included in the article, further inquiries can be directed to the corresponding author.
